# Novel FRET‐Based Biosensors for Real‐Time Monitoring of Estrogen Receptor Dimerization and Translocation Dynamics in Living Cells

**DOI:** 10.1002/advs.202406907

**Published:** 2024-10-17

**Authors:** Kiseok Han, Jung‐Soo Suh, Gyuho Choi, Yoon‐Kwan Jang, Sanghyun Ahn, Yerim Lee, Tae‐Jin Kim

**Affiliations:** ^1^ Department of Integrated Biological Science College of Natural Sciences Pusan National University Busan 46241 Republic of Korea; ^2^ Department of Biological Sciences College of Natural Sciences Pusan National University Busan 46241 Republic of Korea; ^3^ Institute of Systems Biology Pusan National University Busan 46241 Republic of Korea

**Keywords:** biosensors, estrogen receptors (ERs), extracellular matrix, fluorescence resonance energy transfer (FRET), live‐cell imaging

## Abstract

Estrogen receptors (ERs), comprising ER α and ER β, are crucial for regulating cell growth and differentiation via homo‐ and hetero‐dimer formation. However, accurately detecting ER dimerization with precise spatiotemporal resolution remains a significant challenge. In this study, fluorescence resonance energy transfer‐based biosensors to monitor ER dynamics in real‐time, are developed and optimized. This approach involves comprehensive structural analysis, linker comparison, and the selection of optimal fluorescent protein pairs, resulting in three distinct biosensors capable of detecting all ER homo‐ and hetero‐dimerizations within the nucleus. These biosensors are utilized to reveal interactions between ER α/β and calmodulin during dimer formation. Furthermore, by leveraging the ligand‐binding domain (LBD) of ER β, ER ββ LBD biosensor is designed for real‐time analysis of ER ββ homodimerization in the cytoplasm, enhancing the ability to screen ER dimerization‐related drugs. Additionally, we developed a novel ER ββ translocation biosensor, which enables real‐time observation of ER ββ translocation to the nucleus—a capability previously unavailable, is developed. This spatiotemporal analysis demonstrates the relevance of ER translocation in response to drug binding efficacy and extracellular matrix changes. Our biosensors offer transformative tools for studying ER dynamics, providing valuable insights for drug screening and the investigation of ER‐related cellular processes.

## Graphical abstract

Novel FRET‐based estrogen receptor (ER) biosensors are developed through multiple optimization processes to observe the dimerization of ERs. These sensors enable real‐time spatiotemporal analysis of ER α and β homo‐ and heterodimerization in the nucleus and cytoplasm, as well as ER translocation, providing a novel research paradigm for understanding ER dynamics.

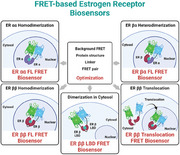

## 1 Introduction

Estrogen receptors (ERs), essential proteins located within the nucleus, play a pivotal role in the regulation of cell growth, differentiation, and survival. They function as the primary mediator of estrogen signaling across various tissues, especially within the female reproductive system.^[^ ^1^ ^]^ ERs have two primary subunits, ER α and ER β, both of which are localized to the cytoplasm. Interaction between subunits can lead to the formation of homodimers (i.e., ER αα or ER ββ) or heterodimers (i.e., ER αβ), each of which can significantly affect cellular physiological responses upon activation.^[^ ^2^ ^]^ The functionality of different ER isoforms is determined by their conserved domains, i.e., the DNA‐binding domain (DBD) and the ligand‐binding domain (LBD). The interaction of ligands with the LBD initiates dimerization, catalyzing the formation of homodimers or heterodimers and the subsequent activation of the ER.^[^ ^3^ ^]^ Once activated, ERs translocate from the cytoplasm to the nucleus via dimerization.^[^ ^4^ ^]^ Within the nucleus, ER dimers interact with estrogen receptor elements (EREs) via DBD, and function as transcription factors that regulate gene expression mediated by EREs.^[^ ^5^ ^]^

Despite the critical role of ER dimerization during sex hormone signaling, real‐time analysis of the dynamics of dimerization poses significant technical challenges. To address these, we developed genetically encoded ER biosensors using fluorescence resonance energy transfer (FRET), a method that facilitates both real‐time observation and the analysis of ER α and ER β dimerization within the nucleus of living cells. FRET, a phenomenon that occurs when two fluorophores pass within 10 nm of each other, allows for the transfer of nonradiative energy and is widely used for detecting protein–protein interactions.^[^ ^6^ ^]^ This technique is particularly advantageous for spatiotemporal analysis since it enables us to overcome previous limitations in studying ER dimerization.^[^ ^7^ ^]^ By implementing a series of targeted optimizations, we significantly enhanced the FRET efficiency of our biosensors. These improvements included linker refinement, selection of the most effective fluorescent protein (FP) pairs, and fine‐tuning of the biosensor sequences. Together, these adjustments led to a marked reduction in background interference, thereby improving the sensitivity and specificity of the biosensor.^[^ ^8^ ^]^

Another challenge involved in characterizing the dynamics of ER dimerization is the lack of tools available to detect and analyze dimerization events within the nucleus and cytosol. This issue is compounded by the tendency of fluorescently tagged ERs to localize primarily to the nucleus due to the presence of a nuclear localization sequence (NLS) situated between the DBD and LBD.^[^ ^9^ ^]^ Although one recent study has explored various ER functions in the cytosol, studies on ER dimerization within this compartment are limited.^[^ ^10^ ^]^ To address this gap, we used the LBD of ER β to develop a FRET‐based biosensor for ER ββ homodimerization capable of detecting dimerization events in the cytoplasm in real time.

Historically, the detection of ER nuclear translocation has relied on methods such as immunostaining or Western blotting, which do not provide real‐time insight.^[^ ^11^ ^]^ Moreover, the large size of genetically encoded FRET biosensors, caused by the attachment of FPs, makes tracking protein translocation within live cells challenging. These constraints limit our capacity for real‐time spatiotemporal analysis of ER translocation. To overcome these hurdles, we developed an ER ββ translocation biosensor capable of the real‐time observation of ER ββ dimerization and nuclear translocation. We hope that this groundbreaking tool may help address challenges in ER dimerization research and pave the way for new discoveries in this field.

## 2 Results

### 2.1 Development of an ER αα Full Length (FL) FRET Biosensor

**Figure 1:**
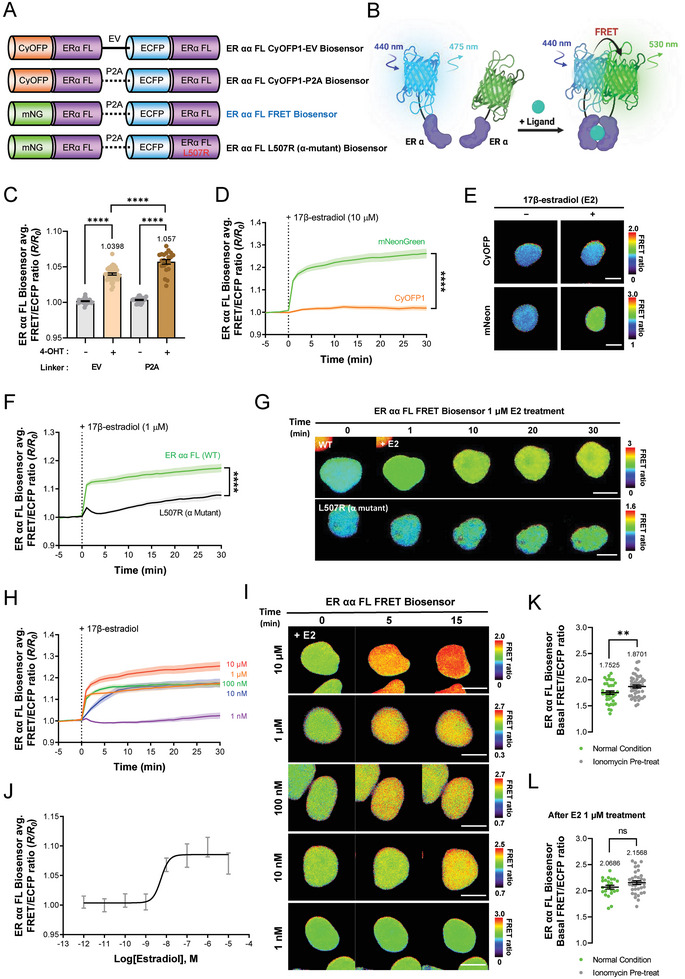
Optimization and Application of the Estrogen Receptor αα Full‐Length (FL) FRET Biosensor. A) Schematic representation and B) mode of action of ER αα FL FRET biosensors. C) Bar graphs depict changes in the normalized FRET/ECFP emission ratio of the ER αα FL CyOFP1‐EV biosensor (EV; n = 39) and the ER αα FL CyOFP1‐P2A biosensor (P2A; n = 21) after 30 min of treatment with 10 µM 4‐hydroxytamoxifen (****p < 0.0001). Mean values are shown above each bar. D) Time course of mean normalized FRET/ECFP emission ratio changes and E) representative images illustrate the FRET ratios of the ER αα FL CyOFP1‐P2A biosensor (CyOFP1; n = 26) and the ER αα FL FRET biosensor (mNeonGreen; n = 27) before and after treatment with 10 µM 17β‐estradiol (****p < 0.0001). The color scale indicates the FRET/ECFP ratio, with warmer and cooler colors representing higher and lower ratios, respectively, scale bar = 10 µm. F) Time course of changes in the mean normalized FRET/ECFP emission ratio and G) representative images depicting FRET ratios for the ER αα FL FRET biosensor (WT; n = 40) and the ER αα FL mutant‐type biosensor (α mutant; n = 35, ****p < 0.0001), scale bar = 10 µm. H) Time course and I) ratio images of changes in the normalized FRET/ECFP emission ratio for ER αα FL FRET biosensors at varying concentrations of 17β‐estradiol (i.e., 10 µM; n = 32, 1 µM; n = 38, 100 nM; n = 34, 10 nM; n = 21, 1 nM; n = 29), scale bar = 10 µm. J) Dose‐dependent effect of 17β‐estradiol on normalized FRET ratio as measured by the ER αα FL FRET biosensor after 6 h of E2 treatment. K) Dot graphs depict the effect of 1 µM ionomycin pretreatment compared with that before (n = 37–49, **p < 0.01) and L) after E2 treatment through changes in the FRET/ECFP basal emission ratio of the ER αα FL FRET biosensor (n = 23–35, ns: not significant). All error bars denote mean (line) ± SEM, and all *p*‐values are derived from Student's *t*‐tests.

To create a FRET biosensor capable of detecting the dimerization of estrogen receptor alpha (ER α), we first used AlphaFold to analyze the proximity between the two terminals of the structure of the ER α homodimer.^[^ ^12^ ^]^ We then attached Cyan‐excitable orange fluorescent protein 1 (CyOFP1) and enhanced cyan fluorescent protein (ECFP) as FRET pairs to the N‐terminal of each ER α monomer (**Table** **S1**; Figure S1A, Supporting Information). CyOFP1 and ECFP were chosen as acceptor and donor FPs,^[^ ^13^ ^]^ respectively, since their overlap integral (J) (i.e., 1.98 λ) can sufficiently induce resonance energy transfer, making them suitable for use as FRET biosensors. Subsequently, we synthesized two versions of the ER αα FL biosensor—one with a CyOFP1‐EV linker and another with a CyOFP1‐P2A linker—that vary only in the linker used to connect the FP‐tagged ER α monomers. We then compared changes in FRET ratio associated with each linker type (**Figure** **1**A). The EV linker acts as a flexible connector between the two ER α monomers, whereas the P2A peptide linker introduces a short sequence of 2A self‐cleaving peptides, which induces ribosomal skipping during translation and allows the two ER αs to exist independently.^[^ ^14^ ^]^

Next, to refine the functionality of our ER biosensor, we initially focused on optimizing the linker component. We subjected two variants of the biosensor—ER αα FL CyOFP1‐EV and ER αα FL CyOFP1‐P2A—to treatment with 17β‐estradiol (E2), a well‐known ER ligand, at a concentration of 10 µM. After observing no notable changes in FRET efficiency 30 min after treatment, we introduced 10 µM of 4‐hydroxytamoxifen (4‐OHT), a compound with a higher affinity than E2 for ER α.^[^ ^15^ ^]^ This led to an observable increase in FRET ratio for both biosensor variants, with the P2A linker variant showing greater FRET efficiency than the EV linker (Figure 1C; Figure S1B, Supporting Information).

We were prompted by the potential for enhanced FRET efficiency as indicated by the broader FRET overlap integral of the mNeonGreen (mNeonG)‐ECFP pair (3.03 λ) relative to the CyOFP1–ECFP pair,^[^ ^16^ ^]^ and therefore CyOFP1 with mNeonG. This adjustment resulted in a significant elevation in FRET efficiency following 30‐min treatment with 10 µM E2, substantiating our hypothesis (Figure 1D,E; Figure S1C, Supporting Information). Based on these systematic optimizations, we then identified the sensor configuration using the P2A linker and mNeonG‐ECFP pair as the optimal ER αα FL FRET biosensor setup (Figure 1A). Figure 1B depicts a schematic illustrating the functionality of the ER αα FL FRET biosensor.

Next, to verify the efficacy of the optimized ER αα FL FRET biosensor, we engineered a variant, named the ER αα FL L507R biosensor, which incorporates site‐directed mutagenesis to impair ER α dimerization.^[^ ^17^ ^]^ Compared to the optimized biosensor, this mutant variant exhibits a notably lower FRET efficiency following a 30‐min exposure to 1 µM of E2, thereby validating its functional improvement (Figures 1F,G; S1D, Supporting Information). Building on the capabilities of the ER αα FL FRET biosensor, we further explored its sensitivity to varying concentrations of E2. This involved assessing FRET efficiency as a function of E2 concentration and enabled the real‐time visualization and quantification of concentration‐dependent FRET reactivity (Figure 1H,I). In addition, we used a microplate reader to determine the detection range slope for the biosensor (Figure 1J). Comprehensive details regarding the calculation of the EC_50_ and the limit of detection (LOD) values are available in **Tables** **S2** and **S3**.

### 2.2 Development of an Estrogen Receptor βα FL FRET Biosensor

**Figure 2:**
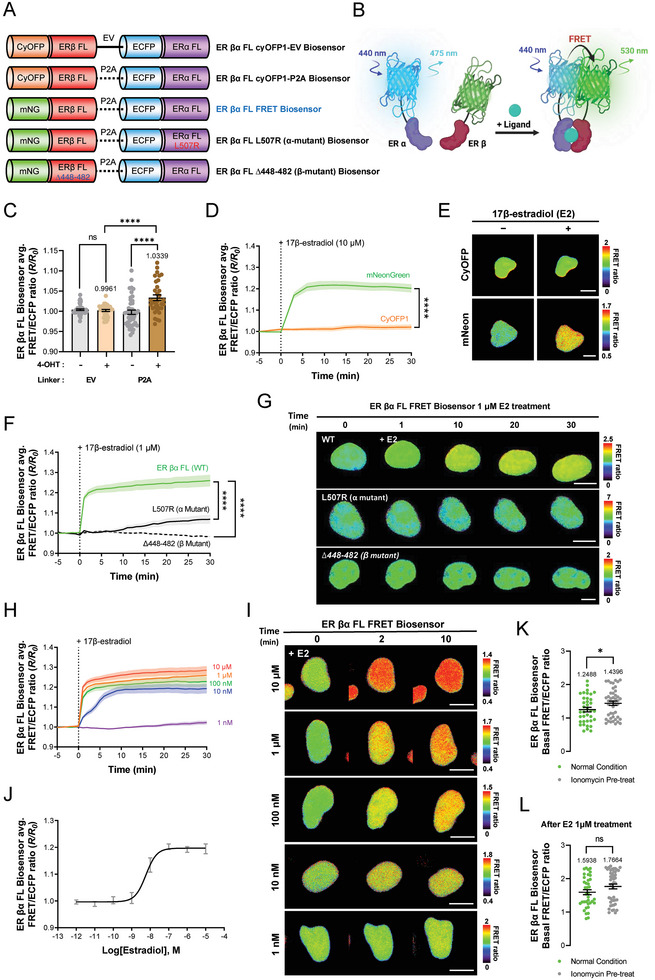
Optimization and Application of the Estrogen Receptor βα Full‐Length (FL) FRET Biosensor. A) Schematic representation and B) mode of action of ER βα FL FRET biosensors. C) Bar graphs depict changes in the normalized FRET/ECFP emission ratio of the ER βα FL CyOFP1‐EV biosensor (EV; n = 33) and the ER βα FL CyOFP1‐P2A biosensor (P2A; n = 33) after 30 min of treatment with 10 µM 4‐hydroxytamoxifen (****p < 0.0001, ns: not significant). D) Time course of changes in the mean normalized FRET/ECFP emission ratio and E) representative images illustrating the FRET ratios of the ER βα FL CyOFP1‐P2A biosensor (CyOFP1; n = 49) and the ER βα FL FRET biosensor (mNeonGreen; n = 14) before and after treatment with 10 µM 17β‐estradiol (****p < 0.0001), scale bar = 10 µm. F) Time course graphs of changes in the mean normalized FRET/ECFP emission ratio and G) representative images showing FRET ratios for the ER βα FL FRET biosensor (WT; n = 24) and ER βα FL mutant‐type biosensors (β mutant; n = 23, α mutant; n = 12, ****p < 0.0001), scale bar = 10 µm. H) Time course and I) ratio images of changes in the normalized FRET/ECFP emission ratio for the ER βα FL FRET biosensors at various concentrations of 17β‐estradiol (i.e., 10 µM; n = 37, 1 µM; n = 24, 100 nM; n = 21, 10 nM; n = 24, 1 nM; n = 14), scale bar = 10 µm. J) Dose‐response curve of 17β‐estradiol on the normalized FRET ratio measured by the ER βα FL FRET biosensor after 6 h of E2 treatment. K) Dot graphs illustrate the effect of 1 µM ionomycin pretreatment compared with that before (n = 37–45, *p < 0.05) and L) after E2 treatment through changes in the FRET/ECFP basal emission ratio of the ER βα FL FRET biosensor. All error bars denote mean (line) ± SEM, and all *p*‐values are derived from Student's *t*‐tests.

Next, the development of the ER βα heterodimerization detection biosensor paralleled the optimization pathway used for the ER αα FL FRET biosensor. Briefly, we initiated this process with a structural analysis (Table S1; Figure S2A, Supporting Information), which led to the construction of two biosensor variants: ER βα FL CyOFP1‐EV, which used the EV linker, and ER βα FL CyOFP1‐P2A, which used the P2A peptide linker. We then compared their FRET efficiencies to determine the optimal linker configuration (**Figure** **2**A). After treating biosensors with E2 and detecting no significant differences in performance, we administered 10 µM of 4‐OHT. After 30 min, the relatively higher FRET efficiency of the P2A linker's relative to the EV linker was easily observable (Figure 2C; Figure S2B, Supporting Information).

We next substituted the acceptor FP with mNeonG and again assessed FRET efficiency following 10 µM E2 treatment. This substitution led to a notable increase in FRET efficiency, verified via live‐cell imaging (Figure 2D,E; Figure S2C, Supporting Information), thereby identifying this configuration as the optimized ER βα FL FRET biosensor. Figure 2B depicts a schematic illustrating the functionality of the ER βα FL FRET biosensor. For further validation, we engineered two mutant biosensors: an ER βα FL L507R (α mutant) created via mutation of the ER α sequence to L507R and an ER βα FL Δ448‐482 (β mutant) created by deleting residues 448–482 in the ER β LBD, which are essential for dimerization since they form helix 11 (Figure 2A).^[^ ^18^ ^]^ Both mutant biosensors displayed significantly reduced FRET efficiency relative to the optimized ER βα FL FRET biosensor following treatment with 1 µM E2 (Figure 2F,G; Figure S2D, Supporting Information).

Subsequently, we examined the performance of the ER βα FL FRET biosensor at varying concentrations of E2, which enabled both real‐time visualization and quantification of concentration‐specific FRET responsiveness (Figure 2H,I). Additionaly, using a microplate reader we determined the detection range slope and calculated the EC_50_, LOD, and limit of quantitation (LOQ) of the biosensor (Figure 2J); all detailed metrics are provided in Tables S2 and S3. This comprehensive approach not only validated the functionality of the optimized biosensor, but also highlighted its ability to detecting ER βα heterodimerization highly specifically and at a high level of sensitivity.

### 2.3 Development of an Estrogen Receptor ββ FL FRET Biosensor

**Figure 3:**
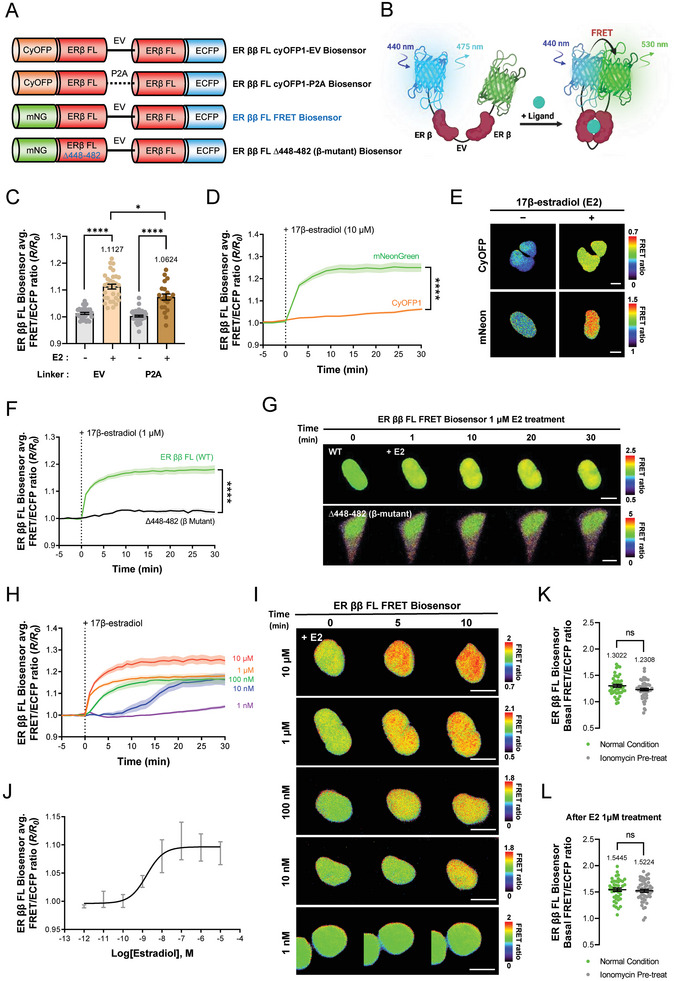
Optimization and Application of the Estrogen Receptor ββ Full‐Length (FL) FRET Biosensor. A) Schematic representation and B) mode of action of ER ββ FL FRET biosensors. C) Bar graphs depict changes in the normalized FRET/ECFP emission ratio between the ER ββ FL CyOFP1‐EV (EV; n = 31) and the ER ββ FL CyOFP1‐P2A biosensors (P2A; n = 21) after treatment with 10 µM 17β‐estradiol for 30 min (****p < 0.0001, *p < 0.05). D) Time course of changes in the mean normalized FRET/ECFP emission ratio and E) representative images illustrating the FRET ratios of the ER ββ FL CyOFP1‐EV (CyOFP1; n = 31) and ER ββ FL FRET biosensors (mNeonGreen; n = 17) before and after treatment with 10 µM 17β‐estradiol (****p < 0.0001), scale bar = 10 µm. F) Time course of changes in the mean normalized FRET/ECFP emission ratio and G) representative images showing FRET ratios for the ER ββ FL FRET (WT; n = 17) and ER ββ FL mutant‐type biosensors (β mutant; n = 31, ****p < 0.0001), scale bar = 10 µm. H) Time course and I) ratio images of changes in the normalized FRET/ECFP emission ratio for the ER ββ FL FRET biosensors at varying concentrations of 17β‐estradiol (i.e., 10 µM; n = 18, 1 µM; n = 27, 100 nM; n = 18, 10 nM; n = 13, 1 nM; n = 39), scale bar = 10 µm. J) Dose‐dependent effect of 17β‐estradiol on normalized FRET ratio as measured by the ER ββ FL FRET biosensor after 6 h of E2 treatment. K) Dot graphs depict the effect of 1 µM ionomycin pretreatment compared with that before (n = 42–52) and L) after E2 treatment through changes in the FRET/ECFP basal emission ratio of the ER ββ FL FRET biosensor. All error bars denote mean (line) ± SEM, and all *p*‐values are derived from Student's *t*‐tests.

To investigate ER ββ homodimerization, we developed two biosensor variants (i.e., ER ββ FL CyOFP1‐EV and ER ββ FL CyOFP1‐P2A); this was accomplished by attaching CyOFP1 to the N‐terminal and ECFP to the C‐terminal of ER β, as guided by structural analysis (Table S1; Figure S3A, Supporting Information). After administering 10 µM of E2 to both biosensors, we noted that the EV linker variant showed a higher FRET efficiency (**Figure** **3**A,C). Following our established optimization protocol, we then replaced the acceptor FP from CyOFP1 with mNeonG, which led to a substantial increase in FRET ratio following treatment with 10 µM E2. This resulted in the identification of this configuration as the optimized ER ββ FL FRET biosensor (Figure 3D,E; Figure S3B, Supporting Information). Figure 3B depicts a schematic illustrating the functionality of the ER ββ FL FRET biosensor.

Next, to validate the functionality of this optimized biosensor, we engineered the ER ββ FL Δ448‐482 biosensor. This variant, which features a deletion mutation in a region crucial for dimerization, exhibited significantly lower FRET efficiency than the wild type after 30 min of exposure to 1 µM E2 (Figure 3F,G; Figure S3C, Supporting Information). Further analyses were then conducted to characterize the ER ββ FL FRET biosensor's responsiveness to E2 concentrations in real time visualization and quantification of concentration‐dependent FRET reactivity (Figure 3H,I). Also, microplate reader was used to perform the calculation of EC_50_, LOD, and LOQ values (Figure 3J), and detailed metrics are provided in Tables S2 and S3.

Making use of meticulous optimization and validation processes, all three FRET‐based ER FL biosensors were eventually confirmed to localize to the nucleus. Their rapid response to E2 and their high sensitivity were cross validated using fluorescence microscopy and measurements taken using a microplate reader, thereby demonstrating detection ranges between 1 and 10 nM and EC_50_ values indicative of their efficacy. Consequently, we conclude that we have successfully developed a suite of biosensors that enable real‐time, spatiotemporal, and quantitative analysis of ER dynamics within the nuclei of living cells. Moreover, this achievement underscores the utility and effectiveness of FRET‐based assays in the detailed study of ERs dynamics.

### 2.4 Identifying Calcium‐Dependent Dynamics of ERs With FRET‐Based ER FL Biosensors

Through a series of optimizations and validations, all three FRET‐based ER biosensors were constructed using the full sequences of ER α and β, allowing them to act intracellularly through the same biological mechanisms as those of the actual ER. Among the ER subunits, only ER α regulates calcium‐dependent gene expression through its interaction with calmodulin (CaM), and the same site on ER α is bound to two lobes of CaM, forming a 1:2 complex, which closes the distance between the two ER αs.^[^ ^19^ ^]^ When the ER αα FL FRET biosensor was pretreated with 1 µM of ionomycin to increase the intracellular calcium concentration, a significant increase in the basal FRET ratio was detected compared with that in the control. Treatment with 1 µM E2 showed no significant difference in FRET ratio compared with that in the ionomycin‐pretreated group after 30 min (Figure 1K,L). These findings confirm that CaM, which is activated by the increase in intracellular calcium concentration through ionomycin, binds to ER α in the ER αα FL FRET biosensor and closes the distance between the two ER α even without E2 treatment, resulting in an increased basal FRET ratio compared with that under normal conditions. Subsequently, treatment with E2 resulted in similar FRET ratios in both groups, suggesting that interaction with CaM does not affect the maximum FRET ratio activity of ER αα homodimerization fully induced by E2. This tendency was also detected in the ER βα FL‐FRET biosensor, wherein there was a significant increase in the basal FRET ratio when the ER βα FL‐FRET biosensor was pretreated with ionomycin compared with that in the untreated group (Figure 2K,L). This could be due to the interaction of CaM with the ER α domain in the ER βα FL‐FRET biosensor, resulting in a conformational change of the ER α portion. Conversely, the ER ββ FL‐FRET biosensor showed no significant difference in the basal FRET ratio compared with that under the normal condition even after treatment with ionomycin because ER β and CaM do not interact with each other (Figure 3K,L). The calcium‐dependent interaction of these ER biosensors is illustrated in detail in Figure S3I (Supporting Information) schematic. These findings demonstrate that the three FRET‐based ER biosensors can be affected by biological regulation mechanisms such as ER in real cells through the full sequence of ER and can be used as a good tool to examine ER dimerization caused by different factors.

### 2.5 Optimization and Application of ER ββ LBD FRET Biosensor

**Figure 4:**
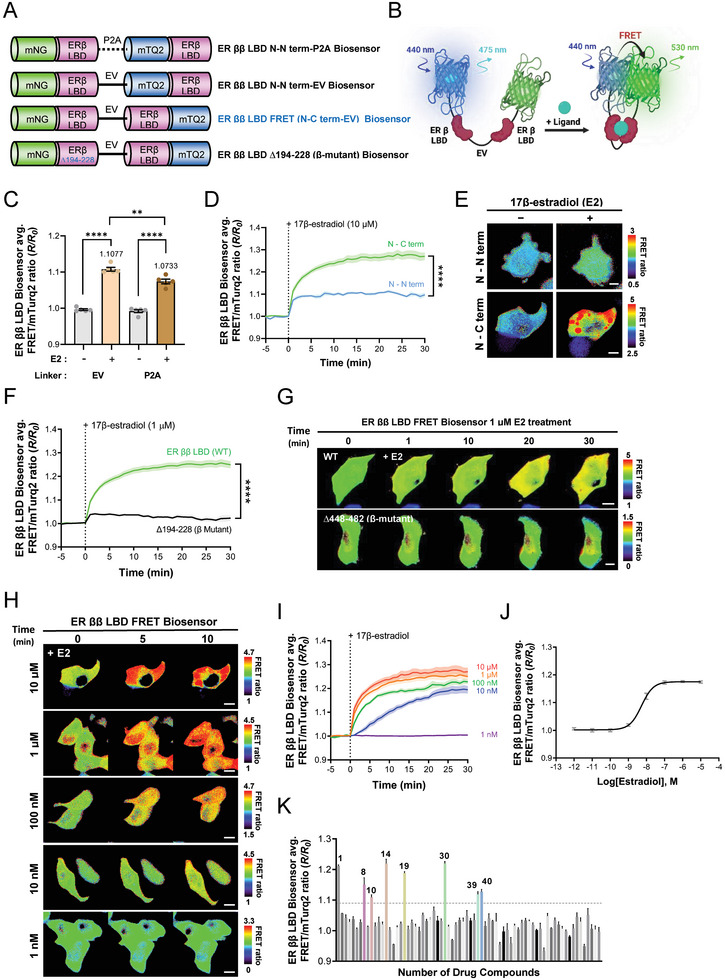
Optimization and Application of the Estrogen Receptor ββ LBD FRET Biosensor. A) Schematic diagram and B) mode of action of ER ββ LBD FRET biosensors. C) Bar graphs depict changes in the normalized FRET/mTurquoise2 emission ratio between the ER ββ LBD N‐N term‐EV (EV; n = 5) and the ER ββ LBD N‐N term‐P2A biosensors (P2A; n = 5) after treatment with 10 µM 17β‐estradiol for 30 min (****p < 0.0001, **p < 0.01). D) Time course of changes in the mean normalized FRET/mTurquoiuse2 emission ratio and E) representative images illustrating the FRET ratios of the ER ββ LBD N‐N term‐EV (N‐N; n = 5) and the ER ββ LBD FRET biosensors (N‐C; n = 13) before and after treatment with 10 µM 17β‐estradiol (****p < 0.0001), scale bar = 10 µm. F) Time course of changes in the mean normalized FRET/mTurquoise2 emission ratio and G) representative images depicting the FRET ratios for the ER ββ LBD FRET (WT; n = 16) and ER ββ LBD mutant‐type biosensors (∆194‐228; n = 9, ****p < 0.0001), scale bar = 10 µm. H) Ratio images and I) time course of changes in the normalized FRET/mTurquoise2 emission ratio for the ER ββ LBD FRET biosensors at various 17β‐estradiol concentrations (i.e., 10 µM; n = 14, 1 µM; n = 17, 100 nM; n = 14, 10 nM; n = 18, 1 nM; n = 11), scale bar = 10 µm. J) Dose‐dependent effect of 17β‐estradiol on normalized FRET ratio measured using the ER ββ LBD FRET biosensor after 6 h of E2 treatment. K) Results of screening 72 compounds using the ER ββ LBD FRET biosensor after six hours (n = 3). The dashed line indicates the EC_50_ value of the ER ββ LBD FRET biosensor, and numbers correspond to target drug numbers. All error bars denote mean (line) ± SEM, and all *p*‐values are derived from Student's *t*‐tests.

To detect ER β homodimerization in both the cytoplasm and nucleus, we designed a FRET‐based biosensor that utilized the LBD of ER β to remove the NLS.^[^ ^20^ ^]^ Based on the established optimization framework, structural analysis confirmed that donor and acceptor FPs conjugated to the N‐termini of both ER β LBDs provided optimal FRET efficiency (Table S1; Figure S4A, Supporting Information). Therefore, we synthesized two variants of the biosensor—i.e., ER ββ LBD N–N term‐P2A and ER ββ LBD N–N term‐EV—by incorporating the mNeonGreen–mTurquoise2 (mTurq2) fluorescent pair (**Figure** **4**A). This pair was chosen due to it having a greater overlap integral than the mNeonGreen–ECFP combination, which suggested a greater potential for FRET efficiency.^[^ ^21^ ^]^ Both variants were found to predominantly reside within the cytoplasm, and upon administration of 10 µM E2, the EV linker variant exhibited significantly greater FRET efficiency than the P2A variant after 30 min (Figure 4C).

However, it was determined that the constructed ER ββ LBD N‐N term‐EV biosensor had an increased background FRET due to the close distance between the donor and acceptor FPs, which may reduce the FRET efficiency even if homodimerization of ER ββ LBD occurs.^[^ ^22^ ^]^ To overcome this limitation, we developed the ER ββ LBD N‐C term‐EV biosensor with enhanced spatial separation between FPs. These modifications resulted in a noticeable improvement in FRET efficiency after 30 min of 10 µM E2 treatment, leading to the identification of this variant as the optimal ER ββ LBD FRET biosensor (Figure 4D,E; Figure S4F, Supporting Information). Figure 4B depicts a schematic illustrating the functionality of the ER ββ LBD FRET biosensor.

We also constructed an ER ββ LBD ECFP biosensor to compare with the mNeonG‐ECFP pair used in ER FL. ER ββ LBD ECFP biosensor exhibited a different FRET behavior when treated with E2, and to overcome this, we constructed ER ββ LBD mECFP by replacing the ECFP to mECFP (Figure S4B,C, Supporting Information). In this case, the FRET ratio increased after 10 µM E2 treatment, but ultimately could not exceed the FRET efficiency level of the ER ββ LBD FRET biosensor using the mNeonG‐mTurq2 pair (Figure S4D,E, Supporting Information).

To validate the action of this optimized biosensor, we constructed an ER ββ LBD Δ194‐228 biosensor. This modification effectively reduced the FRET activity after 1 µM of E2 treatment compared to the optimal sensor and identified this sequence as a critical sequence in the dimerization of ER ββ LBD, highlighting its important role in dimer formation (Figure 4F,G; Figure S4G, Supporting Information). Same as the ER FL biosensors, subsequent experiments performed to determine the biosensor's sensitivity and dynamic range involved analysis of real‐time FRET efficiency analysis across a range of E2 concentrations, thus allowing us to calculate the EC_50_, LOD, and LOQ values (Figure 4H–J); and all detailed metrics are provided in Tables S2 and S3. Interestingly, we observed that cytoplasmic aggregation, which is induced by 10 µM and 1 µM E2 treatments, resulted in significantly higher FRET efficiency within these aggregates compared to areas without aggregation (Figure S4H,I, Supporting Information).

Next, we characterized the properties of the ER ββ LBD FRET biosensor, including high FRET efficiency and good transfection efficiency due to its compact size, to confirm the applicability of the biosensor for drug screening.^[^ ^23^ ^]^ We therefore selected 72 compounds identified as estrogen analogs (Table S4, Supporting Information) and subjected them to equal treatment at a 1 µM concentration in 96‐well plates to assess their ability to induce ER ββ dimerization as measured using FRET ratios. Using serial dilution of E2, we identified the detection range slope of the biosensor and employed the calculated EC_50_ value as a baseline to evaluate which of the tested drugs were capable of inducing ER ββ dimerization. Our findings revealed that, excluding E2 (Drug #1) itself, seven drugs demonstrated FRET ratios surpassing the EC_50_ value of E2, which suggests that they have the potential to induce dimerization (Figure 4K). The seven drugs including E2 detected by the drug screen were cross validated using fluorescence microscopy, which showed a similar pattern of FRET efficiency increase to that of E2 in real time (Figure S5A, Supporting Information), and significant increases in FRET efficiency were observed after 30 min for all drugs (Figure S5B–J, Supporting Information). We therefore concluded that the development of the ER ββ LBD FRET biosensor for the cytoplasmic detection of ER β dimerization, based on the LBD of ER β, has utility for live‐cell drug screening applications. The biosensor's rapid and sensitive response to E2 and its ability to identify compounds that promote ER ββ dimerization showcase the potential of FRET‐based assays for the study of ER dynamics and related drug discovery processes.

### 2.6 Real‐Time Tracking of the Cytoplasm‐to‐Nucleus Translocation of ERs

**Figure 5:**
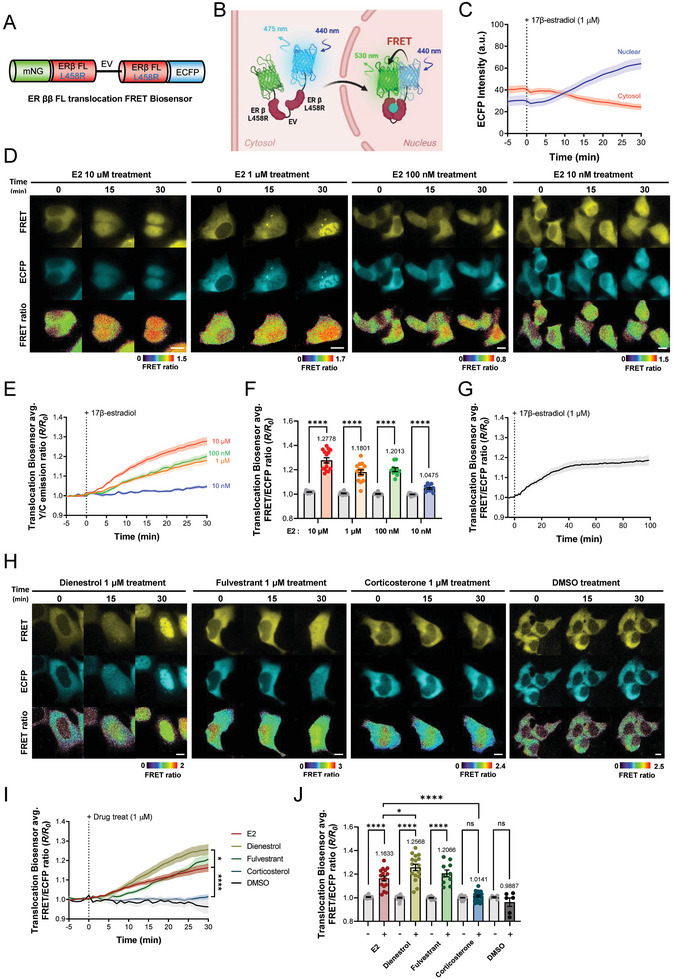
Validation of the Estrogen Receptor ββ FL Translocation FRET Biosensor. A) Schematic representation and B) mode of action of the ER ββ FL translocation FRET biosensor. C) Time course of changes in the normalized ECFP fluorescence intensity in the cytosol (n = 16) and nucleus (n = 19) before and after treatment with 1 µM 17β‐estradiol. D) Ratio images illustrating FRET, ECFP, and changes in the normalized FRET/ECFP emission ratio and E) time course and F) bar graphs of changes in the normalized FRET/ECFP emission ratio of the ER ββ FL translocation FRET biosensor before and after treatment with 17β‐estradiol (10 µM; n = 15, 1 µM; n = 14, 100 nM; n = 10, 10 nM; n = 13), scale bar = 10 µm. G) Time course of changes in the mean normalized FRET/ECFP emission ratio of the ER ββ LBD translocation FRET biosensor before and after treatment with 1 µM 17β‐estradiol for 100 min (n = 12). H) Ratio images, I) time course, and J) bar graphs of the ER ββ FL translocation FRET biosensor before and after treatment with various drugs at 1 µM (E2; n = 16, dienestrol; n = 16, fulvestrant; n = 10, corticosterone; n = 28, ****p < 0.0001, *p < 0.05), scale bar = 10 µm. All error bars denote mean (line) ± SEM, and all *p*‐values are derived from Student's *t*‐tests.

In this study, we used ER ββ FL FRET and ER ββ LBD FRET biosensors to understand the dynamics of ER ββ, particularly with respect to its homodimerization in the nucleus and cytoplasm. However, one of the main challenges was to accurately track and analyze the intracellular compartmental translocation of ER β, which is essential for the regulation of gene expression. To overcome this limitation, we developed a novel ER ββ FL FRET biosensor that can move from the cytoplasm to the nucleus, namely the ER ββ FL translocation FRET biosensor (**Figure** **5**A). Figure 5B depicts a schematic illustrating the functionality of the ER ββ FL translocation FRET biosensor.

Since ER β—unlike its counterpart ER α, lacks a dimerization‐inhibiting mutation—we incorporated an L458R mutation into its sequence. This was achieved via site‐directed mutagenesis, as per the procedure used for the L507R mutation introduced into the mutant ER αα FL FRET biosensor. In real‐time live cell imaging, we observed that the expression of this biosensor is located in the cytoplasm, which is different from the existing ER ββ FL biosensor, which is located in the nucleus. Notably, following the application of 1 µM E2, the biosensor demonstrated a progressive increase in FRET ratio as it migrated into the nucleus (Figure 5D). To understand the spatiotemporal dynamics of this translocation, we separately measured ECFP intensities in the cytosol and nucleus. We discovered that nuclear ECFP intensity began to increase approximately five minutes after treatment with E2, with a notable reversal of cytosolic and nuclear intensities after 10 min (Figure 5C). This behavior, which did not occur in response to DMSO treatment, highlighted the biosensor's specificity for E2 as well as its ability to translocate into the nucleus, thereby enhancing FRET (Figure 5H).

In further experiments with varying concentrations of E2, ER ββ homodimers were consistently translocated to the nucleus within 30 min after treatment with 10 nM E2 concentration or higher using real‐time imaging (Figure 5D). This allowed for a detailed quantitative analysis of E2‐induced ER ββ dimerization, as indicated by the increased FRET ratio observed in the nuclei at all concentrations tested (Figure 5E,F). We also analyzed the maximum FRET efficiency (FRETmax) when treated with 1 µM of E2 and found a t_1/2_ value of 11 min, reaching a FRETmax of 18% after 40 min of E2 treatment and then maintaining a constant value (Figure 5G). Taken together, these results demonstrate that the ER ββ FL translocation FRET biosensor has the advantages of high sensitivity to E2, the ability to detect concentrations above 10 nM, and the ability to provide real‐time spatiotemporal analysis of the rapid translocation to the nucleus after E2 binding.

### 2.7 Correlating the ER β Binding Affinity of Drugs with ER Translocation Using an ER ββ FL Translocation FRET Biosensor

Next, we investigated the correlation between the binding affinity of drugs to ER β and their ability to induce ER translocation to the nucleus.^[^ ^15^, ^24^ ^]^ To do so, we used the ER ββ FL translocation FRET biosensor for a series of experiments in which we screened drugs exhibiting varying affinities for ER β. To compare responsiveness to the biosensor, we treated it with 1 µM of fulvestrant, a drug known to competitively inhibit the ER in addition to E2 and with similar binding affinity for ER β as E2. Real‐time imaging showed that 30 min after treatment, fulvestrant also bound to the biosensor, translocated to the nucleus, and induced FRET activity to a similar extent as E2 (Figure 5H–J). Furthermore, we explored the effects of dienestrol, a compound with a binding affinity for ER β approximately four times greater than that of E2, and corticosterone, known to show minimal ER β affinity. Comparing FRET ratios using real‐time imaging at a dose of 1 µM of each compound, dienestrol could be observed to bind to the biosensor and translocate to the nucleus, with a significantly higher FRET ratio than E2 30 min after drug treatment. Corticosterone, conversely, could not be seen translocating to the nucleus within the same time and did not cause a significant change in FRET efficiency (Figure 5H–J). Taken together, measurements using the ER ββ FL translocation FRET biosensor suggest that there is a direct relationship between the binding affinity of drugs to ER β and their translocation kinetics, including FRET efficiency. Furthermore, the results highlighted the utility of being able to spatiotemporally analyze drug interactions with ER β in real time.

### 2.8 Regulation of ER Translocation by Extracellular Matrix Stiffness as Measured Using the ER ββ FL Translocation FRET Biosensor

The stiffness of the extracellular matrix (ECM) plays a pivotal role in cellular mechanics and gene regulation since it impacts both the structural integrity of the nucleus and chromatin configuration, both of which in turn influence gene expression patterns.^[^ ^25^ ^]^ Moreover, ECM stiffness is also known to affect the dimensions of the nuclear pore complex, which is crucial for nuclear–cytoplasmic shuttling of transcription factors.^[^ ^26^ ^]^ In cancer, cells are often found within an ECM that is markedly stiffer than normal, largely due to the remodeling activities of cancer‐associated fibroblasts. This difference can be starkly illustrated by comparing the ECM stiffness of normal versus cancerous breast tissue, with normal breast tissue exhibiting an ECM stiffness of ≈0.1 kPa, whereas breast cancer tissue presents a significantly higher stiffness of 4–5 kPa.^[^ ^27^ ^]^

**Figure 6:**
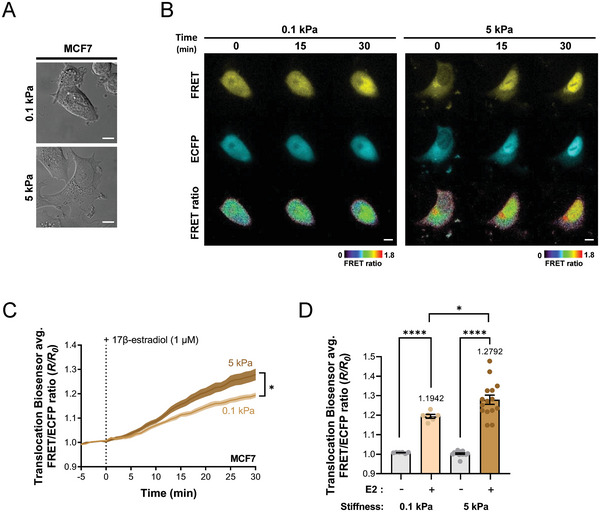
ECM Stiffness Affects the Nuclear Translocation and FRET Activities of the ER ββ FL Translocation FRET Biosensor. A) Scanning DIC microscopy images of MCF‐7 cells on collagen I coated gels with varying stiffness. B) Time course images of FRET, ECFP, and FRET ratio, C) time courses, and D) dot graphs illustrate changes in mean normalized FRET/ECFP emission ratio for the ER ββ FL Translocation FRET biosensor before and after treatment with 1 µM 17β‐estradiol for 30 min (5 kPa; n = 15, 0.1 kPa; n = 6, ****p < 0.0001, *p < 0.05), scale bar = 10 µm. All error bars represent SEM, and all *p*‐values are derived from Student's *t*‐tests.

Here, we investigated how ECM stiffness influences ER β dynamics. To do so, MCF7 cells transfected with the ER ββ FL translocation FRET biosensor were cultured on gels designed to mimic the ECM stiffness of both normal and cancerous breast tissue. The observed morphological differences were significant; cells on the 0.1 kPa gel maintained a rounded morphology, whereas those on the 5 kPa gel adopted a more spread‐out morphology (**Figure** **6**A). Real‐time imaging demonstrated by enhanced fluorescence intensity and FRET ratio that nuclear translocation of ER ββ homodimers was promoted within 30 min after treatment with 1 µM E2 under both 0.1 kPa and 5  kPa conditions (Figure 6B). Quantitative analysis of these results showed that the FRET ratio was significantly higher at 5 kPa than at 0.1 kPa 30 min after E2 treatment, indicating an obvious correlation between ECM stiffness and the efficiency of ER β translocation (Figure 6C,D). This suggests that high ECM stiffness, a hallmark of cancer tissue, may amplify ER translocation, leading to higher ER‐mediated gene expression within cancer cells compared to normal cells. It also highlights that in cells that endogenously express the ER, mechanical signals from the ECM can influence cell behavior and gene expression.

## 3 Discussion

Numerous studies have focused on optimizing linkers and FP pairs for FRET to enhance the efficiency of genetically encoded FRET biosensors.^[^ ^8^, ^28^ ^]^ In this paper, we aimed to design more efficient FRET‐based biosensors by conducting a structural analysis of ER dimers using AlphaFold. Using the optimization processes that compared FRET efficiencies by varying linkers and FP pairs, we developed three FRET biosensors for ER αα, βα, and ββ FL. We showed that these biosensors are highly sensitive, rapidly respond to E2, exhibit activity levels akin to the natural ER by using the FL ER and can spatiotemporally analyze ER dynamics within the nucleus. Moreover, all three biosensors quickly detected E2 in both HeLa and MCF7 cells (Figures S1E–I, S2E–I, S3D–H, Supporting Information), two ER‐positive cell lines commonly used to study ER‐related diseases,^[^ ^29^ ^]^ thereby highlighting the potential of these three biosensors for a wide range of applications related to researching ER‐related conditions.

Furthermore, the ER ββ LBD FRET biosensor was developed specifically to facilitate the screening of drugs that induce ER ββ dimerization in the cytoplasm in real time. This was facilitated by its high sensitivity and rapid response to E2 and the selective use of LBD to minimize intrinsic signal disturbances from other domains that can affect cellular metabolic processes. Our screening of 72 drugs identified eight potentially capable of inducing ER ββ dimerization. Of these, meso‐hexestrol (drug #8), diethylstilbestrol (#14), and 17α‐ethinyl estradiol (#19) were noted as competitive inhibitors of E2,^[^ ^30^ ^]^ whereas coumestrol (#30), daidzein (#39), and genistein (#40) were identified as phytoestrogens, i.e., substances derived from plants that can bind to ERs.^[^ ^31^ ^]^ Moreover, estrone (#10) was recognized as an endogenous estrogen.^[^ ^32^ ^]^ Taken together, these findings emphasize the usefulness of the ER ββ LBD FRET biosensor as a tool for validating ER dimerization‐modulating drugs and illustrate an important application: drug discovery screening.

In addition, in this study we also introduced and validated the ER ββ FL translocation FRET biosensor that can closely mimic the mechanism of regular ER β. This biosensor detects real‐time translocation processes by altering a single amino acid in the ER β sequence, allowing it to locate and mimic the behavior of natural ER. The L458R mutation in ER β, which is analogous to L507 in ER α,^[^ ^17a^ ^]^ suggests that this alteration may conceal the NLS and cause the ER to reside initially in the cytoplasm. Next, upon ligand binding to the LBD, a conformational change may reveal the NLS, thereby leading to nuclear translocation. However, this hypothesis requires further investigation in future studies. Nonetheless, our data show that the binding affinity of drugs to ER β can influence the translocation of the ER β homodimer, thereby opening avenues for research into the correlation between drug binding affinity and nuclear translocation targeting ER. We also present novel evidence that ECM stiffness affects ER ββ dimer translocation and dimerization, thereby linking ER‐related diseases to ECM.

In summary, this work introduces a novel and intuitive way to study ERs by providing real‐time spatiotemporal analysis of ER dimerization dynamics and the translocation across the nuclear and cytoplasmic compartments of living cells using five distinct ER FRET biosensors. These tools have the potential to develop new therapies targeting ER dimerization and for advancing the study and therapy of ER‐related disease.

## 4 Experimental Section

### Computational Protein Structure Prediction

The FL structures of the ER α and ER β monomers were retrieved from the AlphaFold Protein Structure Database (AFDB) using the codes AF‐P03372 and AF‐Q92731, respectively. The structure of the ER β LBD dimer was acquired from the Protein Data Bank using the identifier 5TOA. Next, an alignment tool available in PyMol was used to reconfigure the ER LBD dimer and the ER FL dimer.

### DNA Plasmid Construction

The construction of the Estrogen Receptor Full Length (ER FL) biosensors was conducted through sequential processes that involved the amplification of individual domains by PCR, followed by digestion and ligation, as depicted in Figure 1. Accordingly, the full ER α sequence was first amplified from pEGFP‐C1‐ER α (Addgene plasmid #28230) and digested with XhoI/BspEI or NheI/SalI. Similarly, the full ER β sequence was obtained from pEGFP‐C1‐ER β (Addgene plasmid #28237) and digested with XhoI/BspEI or MluI/NotI. The acceptor FPs CyOFP1 and mNeonGreen were then amplified from pKK‐TEV‐CyOFP1 (Addgene plasmid #105799) and ER‐mNeonGreen (Addgene plasmid #13704), respectively, before being digested with BamHI/XhoI to generate FP FRET pairs. The donor FP ECFP was then amplified from Eevee‐ROCK (provided by the lab of Michiyuki Matsuda) and digested with NotI/XbaI. Next, the EV linker and P2A peptide sequences were amplified from pGEMT‐TPE2A‐Mef2c‐Tdtomato‐Gata4‐Tbx5 (Addgene plasmid #111818) and pCAGGS‐6011nes (Addgene plasmid #108652) before being digested with BspEI/MluI. Next, to construct the ER ββ LBD FRET biosensor, the ER β LBD domain was specifically amplified by PCR from the LBD portion of pEGFP‐C1‐ER β using NsiI/KpnI or BspEI/AflII. The acceptor FP mNeonGreen was then obtained from ER‐mNeonGreen and cut with NheI/NsiI. The donor FPs, ECFP and mTurquoise2, were amplified from Eevee‐Rock and pLL3.7m‐mTurquoise2‐SLBP(18‐126)‐IRES‐H1‐mMaroon1 (Addgene plasmid #83842), respectively, and were cut using AvrII/AflII. Subsequently, the EV linker and P2A peptides were amplified from the same plasmids as those used for the ER FL FRET biosensor before being digested with KpnI/BspEI. Estrogen receptor mutants, including L507R (α mutant) in the ER αα and βα FL FRET biosensors, ∆448‐482 (β mutant) in the ER βα and ββ FL FRET biosensors, and ∆194‐228 (β mutant) in a ER ββ LBD FRET biosensor, were also analyzed. Each of these were created via deletions using site‐directed mutagenesis. In addition, a ER ββ LBD ECFP biosensor was also generated via A206K site‐directed mutagenesis, resulting in an ER ββ LBD mECFP biosensor (Figure S4B, Supporting Information). SnapGene software was used for simulation and guidance throughout all cloning and site‐directed mutagenesis procedures.

### Cell Culture and Transient Transfection

LentiX‐293T cells (Clontech, 632180), HEK293A cells (obtained from the lab of Jihye Seong), HeLa cells (Korea Cell Line Bank, 10002), and MCF‐7 cells (Korea Cell Line Bank, 30022) were cultured in Dulbecco's Modified Eagle Medium (DMEM, GenDEPOT, CM002) enriched with 10% (v/v) fetal bovine serum (FBS, Gibco, 16000–044) and 1% (v/v) of a 100x penicillin–streptomycin solution (i.e., 100 units/mL of penicillin and 100 µg mL⁻^1^ of streptomycin, GenDEPOT, CA005). All cells were maintained in a humidified environment at 5% CO_2_ and at 37 °C in covered, glass‐bottomed confocal dishes (SPL, 200350). For transfection, DNA plasmids were introduced into all cells using a PEIpro transfection reagent (Polyplus), with all procedures following the manufacturer's instructions.

### Microscopy and Image Acquisition

Cells were grown in confocal dishes (SPL, 200350) and starved in DMEM supplemented with 0.5% (v/v) FBS for 9–12 h before imaging. Before all experiments, cells were rinsed with PBS (WELGENE, LB004‐02) and the medium was replaced with a CO_2_‐independent medium (Gibco, 18045–088) containing 0.5% FBS, 1% (v/v) penicillin–streptomycin solution (GenDEPOT, 100X, CA005), and 1X GlutaMAX (Gibco, 35050–061). Imaging was then performed using a Leica DMi8 fluorescence microscope equipped with an LED8 light source, an HC PL APO 40/1.30 oil immersion objective, a DIC module, a K5‐sCMOS camera, and a CO_2_/37 °C incubation chamber. For ECFP, mTurquoise2, and FRET imaging, a 436/20 excitation filter, a 455 dichroic mirror, and two different emission filters (i.e., 480/40 for ECFP and 535/30 for FRET) were used. Background removal and image capture procedures for the ECFP, FRET, and FRET ratio modes were performed using LAS X.

### Optimizing FP FRET Pairs and FRET Images

To evaluate the FRET efficiencies of FP pairs, the FRET overlap integral protocol described by FPbase (https://www.fpbase.org/) was used. The equation employed is as follows:

(1) $$J\left(\lambda \right)=\int F_{D}\left(\lambda \right)\;\times \;E_{A}\left(\lambda \right)\;\times \;\lambda^{4}d\lambda$$

Here, *J*(λ) denotes the FRET overlap integral and incorporates both the peak‐normalized donor fluorescence spectra *F_D_* (λ) and the acceptor fluorescence excitation spectra *E_A_* (λ).^[^ ^33^ ^]^ Images were captured and fluorescence emission intensities were computed using LAS X version 3.6.0 (Leica, https://www.leica‐microsystems.com/products/microscope‐software/p/leica‐las‐x‐ls/). A specific region of interest (ROI) was selected to monitor signals and conduct quantification. The fluorescence intensity of a designated background region was then quantified and subtracted from ROI signals generated by the fluorescence channels. The FRET ratio, which is based on the background‐subtracted fluorescence intensity images of FRET and ECFP (i.e., mTurquoise2), was calculated pixel‐by‐pixel using the following formula:

(2) $$FRET\;ratio=\frac{FRET_{ROI}\;Em.\;-\;FRET_{background}\;Em.}{CFP_{ROI}\;Em.\;-\;CFP_{background}\;Em.}$$

Here, Em. indicates the emission intensity of each filter. Ratio images were visualized in intensity‐modified display mode, with pixel color reflecting the FRET/CFP intensity ratio.

### Chemicals and Drug Compounds

17β‐Estradiol (Selleck Chem, S1709), 4‐Hydroxytamoxifen (Selleck Chem, S7827), Fulvestrant (Selleck Chem, S1191), Dienestrol (Selleck Chem, S1858), corticosterone (Selleck Chem, S4752) and ionomycin (Sigma‐Aldrich, I9657) solutions were all prepared in DMSO (Biosesang, AC4002‐050‐00). A total of 72 estrogen analogs were acquired from the Ministry of Food and Drug Safety of the Republic of Korea, with each analog also diluted in DMSO.

### Microplate Reader‐Based FRET Assays and Subsequent Measurements

To investigate the unique characteristics of the biosensors, ER FRET biosensors were transfected into HEK293A cells within 75T flasks (SPL, 70075). 24 h after transfection, cells were harvested and resuspended in clear DMEM (GenDEPOT, CM004‐310) containing 10% FBS, 1% penicillin (100 units/mL), and streptomycin (100 µg mL⁻^1^). Next, 3,500 cells were allocated to each well of a clear‐bottomed 96‐well black plate (Greiner Bio‐One, 655087). To control for background FRET signals, untransfected cells were placed in a single column of a 96‐well black plate and incubated overnight. A serial dilution of E2, ranging from 10 µM to 1 pM in final concentration, was added to each well. FRET signals were recorded using a CLARIOStar Plus microplate reader (BMG Labtech) at 37 °C, with fluorescence measurements being taken at 433–20/480–20 nm (i.e., the ECFP Excitation/Emission wavelength) and 433–20/520–20 nm (i.e., the FRET Excitation/Emission wavelength). Plates were evaluated at 1, 6, and 24 h after treatment. The FRET ratio was calculated as follows:

(3) $$FRET\;ratio=\frac{FRET_{transfected\;cell}-mean\;FRET_{untransfected\;cell}}{CFP_{transfected\;cell}-mean\;CFP_{untransfected\;cell}}$$

Following a similar protocol, the ER ββ LBD FRET biosensor was transfected into HEK293A cells in 75‐T flasks to screen 72 estrogen analogs. Cells were collected 24 h after transfection, and 3 500 cells were then distributed into each well of a clear‐bottom 96‐well black plate. In addition, untransfected cells were then placed in three wells of a 96‐well black plate and incubated overnight. Wells were treated with E2 and estrogen analogs at a final concentration of 1 µM. Readings were then taken at 1, 6, and 24 h after treatment using a CLARIOStar Plus microplate reader at 37 °C.

### Preparation of Polyacrylamide Gels

The methods used to prepare and characterize gels with varying elastic moduli are described in a previous study.^[^ ^34^ ^]^ This process involved casting polyacrylamide (PA) gels onto glass‐bottom dishes that had been sequentially coated with 3‐aminopropyltrimethoxysilane (Sigma‐Aldrich, 281778) and glutaraldehyde (Junsei, 27372‐1230). Next, stock solutions of 40% acrylamide (Bio‐Rad, 161‐0140) and 2% bis‐acrylamide (Bio‐Rad, 161‐0142) were used to introduce variation in gel stiffness. The final concentrations of PA solution (5%) and the bis‐acrylamide crosslinker (i.e., 0.009% and 0.14%) were then adjusted to achieve gel stiffness values of 0.1 kPa (soft) and 5 kPa (stiff), respectively. PA polymerization was then initiated by adding 2.5 µL of 10% w/v ammonium persulfate (TransLab, TLP‐109.1) and 0.25 µL of N,N,N′,N′‐tetramethylethylenediamine (Biosesang, T1004). Subsequently, 10 µL of this solution was dispensed into each dish. To enhance cell attachment, sulfosuccinimidyl 6(4′‐azido‐2′‐nitrophenylamino)hexanoate (Thermo, 22589), a photoactive crosslinker, was applied to the gel surface in 100 mM HEPES (Dojindo, GB10). Finally, 200 µL of a 10 µg mL⁻^1^ collagen I solution was added to the PA gels and the dishes were incubated overnight at 37 °C.

### Statistical Analysis

All measurements were expressed as the mean ± standard error of the mean all data were analyzed using GraphPad Prism 9.0. Statistical differences between the means of two groups were evaluated using unpaired two‐tailed *t*‐tests. The level of statistical significance of differences in mean was determined using by *p*‐values, denoted here as follows: *p < 0.05, **p < 0.01, ***p < 0.001, and ****p < 0.0001.

## Conflict of Interest

The authors declare no conflict of interest.

## Author Contributions

K.H. and T.‐J.K. initiated the project. K.H., J.‐S.S., Y.‐K.J., S.A., and Y.L. performed ER FRET biosensors synthesis. G.C. solved computational protein structure prediction. K.H., G.C., Y.‐K.J., and Y.L. performed cell and microscopy experiments. K.H. and G.C. performed microplate reader screening experiments. K.H. processed and analyzed the data. T.‐J.K. supported and supervised the project. K.H. and T.‐J.K. wrote the manuscript.

## Supporting information



Supporting Information

## Data Availability

The data that support the findings of this study are available from the corresponding author upon reasonable request.
