# Supplementary material for: Novel FRET‐Based Biosensors for Real‐Time Monitoring of Estrogen Receptor Dimerization and Translocation Dynamics in Living Cells
Source: Adv Sci (Weinh). 2024 Oct 17;12(1):2406907. doi: 10.1002/advs.202406907 (PMC11714219; doi:10.1002/advs.202406907)
Supplement: Supplementary file 1 — Supporting Information [file ADVS-12-2406907-s001.pdf]

ADVANCED SCIENCE
Open Access

Supporting Information

for *Adv. Sci.*, DOI 10.1002/advs.202406907

Novel FRET-Based Biosensors for Real-Time Monitoring of Estrogen Receptor Dimerization and Translocation Dynamics in Living Cells

*Kiseok Han, Jung-Soo Suh, Gyuho Choi, Yoon-Kwan Jang, Sanghyun Ahn, Yerim Lee and Tae-Jin Kim**

Supporting Information

Novel FRET-based Biosensors for Real-time Monitoring of Estrogen Receptor Dimerization and Translocation Dynamics in Living Cells

Kiseok Han, Jung-Soo Suh, Gyuho Choi, Yoon-Kwan Jang, Sanghyun Ahn, Yerim Lee, and Tae-Jin Kim*

Table S1. Distance values between the two ends of the ER.

**Full Length (FL)**						**Ligand Binding Domain (LBD)**	
**ER α–ER α**	**Distance (nm)**	**ER β–ER α**	**Distance (nm)**	**ER β–ER β**	**Distance (nm)**	**ER β–ER β**	**Distance (nm)**
N–N	5.3	N–N	2.55	N–N	7.55	N–N	2.43
C–C	11.92	C–C	10.24	C–C	6.26		
$N_{\alpha1}$–$C_{\alpha2}$	8.75	N_{α}–C_{β}	6.49	$N_{\beta1}$–$C_{\beta2}$	1.85	C–C	7.12
$C_{\alpha1}$–$N_{\alpha2}$	8.75	C_{α}–N_{β}	5.33	$C_{\beta1}$–$N_{\beta2}$	1.96		

Table S2. Comparison of ER FRET biosensor specificity measurements taken via fluorescent microscopy.

Biosensor name	FRET pair	Linker	Detection range	Kinetics	10 µM	1 µM	100 nM	10 nM	1 nM
**ER αα FL FRET**	mNeonGreen ECFP	P2A	10 nM–1 nM	**FRETmax**	1.231	1.151	1.164	1.167	1.025
				**$T_{1/2}$ (min)**	1.207	0.708	1.038	3.479	22.32
**ER βα FL FRET**	mNeonGreen ECFP	P2A	10 nM–1 nM	**FRETmax**	1.267	1.238	1.217	1.192	1.021
				**$T_{1/2}$ (min)**	0.729	0.759	0.803	3.348	19.52
**ER ββ FL FRET**	mNeonGreen ECFP	EV	10 nM–1 nM	**FRETmax**	1.247	1.172	1.162	1.164	1.042
				**$T_{1/2}$ (min)**	1.903	1.580	3.927	14.84	22.56
**ER ββ LBD FRET**	mNeonGreen mTurquoise2	EV	10 nM–1 nM	**FRETmax**	1.263	1.244	1.206	1.198	1.005
				**$T_{1/2}$ (min)**	2.058	2.281	2.731	9.246	23.32

Table S3. Comparison of ER FRET biosensor specificity measurements taken via microplate reader.

Biosensor Name	$LogEC_{50}$ (M)	EC_{50} (nM)	LOD (nM)	LOQ (nM)	FRETmax
**ER αα FL FRET**	−8.261	5.48	12.6	–	1.085
**ER βα FL FRET**	−8.235	5.82	0.47	33.9	1.201
**ER ββ FL FRET**	−8.785	1.64	1.91	3.24	1.097
**ER ββ LBD FRET**	−8.273	5.33	1.66	7.76	1.175

Table S4. A list of 72 Different Drugs.

No.	Drug Name	No.	Drug Name	No.	Drug Name
1	17β-Estradiol (E2)	25	Procymidone	49	Flavone
2	5α-Dihydrotestosterone	26	Linuron	50	Fluoranthene
3	Bisphenol A	27	Kaempferol	51	Propylthiouracil
4	Progesterone	28	Bisphenol B	52	Sodium azide
5	4-Cumylphenol	29	p,p'-Methoxychlor	53	L-Thyroxine
6	Di-n-butyl phthalate	30	Coumestrol	54	Fenarimol
7	4-tert-Octylphenol	31	Hydroxyflutamide	55	Nilutamide
8	Meso-Hexestrol	32	Cyproterone acetate	56	Actinomycin D
9	Ethyl paraben	33	Ketoconazole	57	4-Hydroxytamoxifen
10	Estrone	34	Apigenin	58	ICI 182,780
11	Medroxyprogesterone acetate	35	Dexamethasone	59	Mifepristone
12	Corticosterone	36	Phenolphthalin	60	Pimozide
13	4-Androstenedione	37	Cycloheximide	61	12-O-Tetradecanoylphorbol-13-acetate
14	Diethylstilbestrol	38	Testosterone	62	Zearalenone
15	Flutamide	39	Daidzein	63	Anastrazole
16	Haloperidol	40	Genistein	64	Dibenzo[a,h]anthracene
17	2-sec-Butylphenol	41	Diethylhexyl phthalate	65	Apomorphine
18	Vinclozolin	42	17β-Trenbolone	66	Norethynodrel
19	17α-Ethinyl estradiol	43	Bicalutamide	67	p-n-Nonylphenol
20	Reserpine	44	Methyl testosterone	68	Tamoxifen
21	Spironolactone	45	Clomiphene citrate	69	Morin
22	Butylbenzyl phthalate	46	p,p'-DDE	70	Fadrozole
23	Atrazine	47	o,p'-DDT	71	Kepone (Chlordecone)
24	17α-Estradiol	48	Finasteride	72	Methyltrienolone

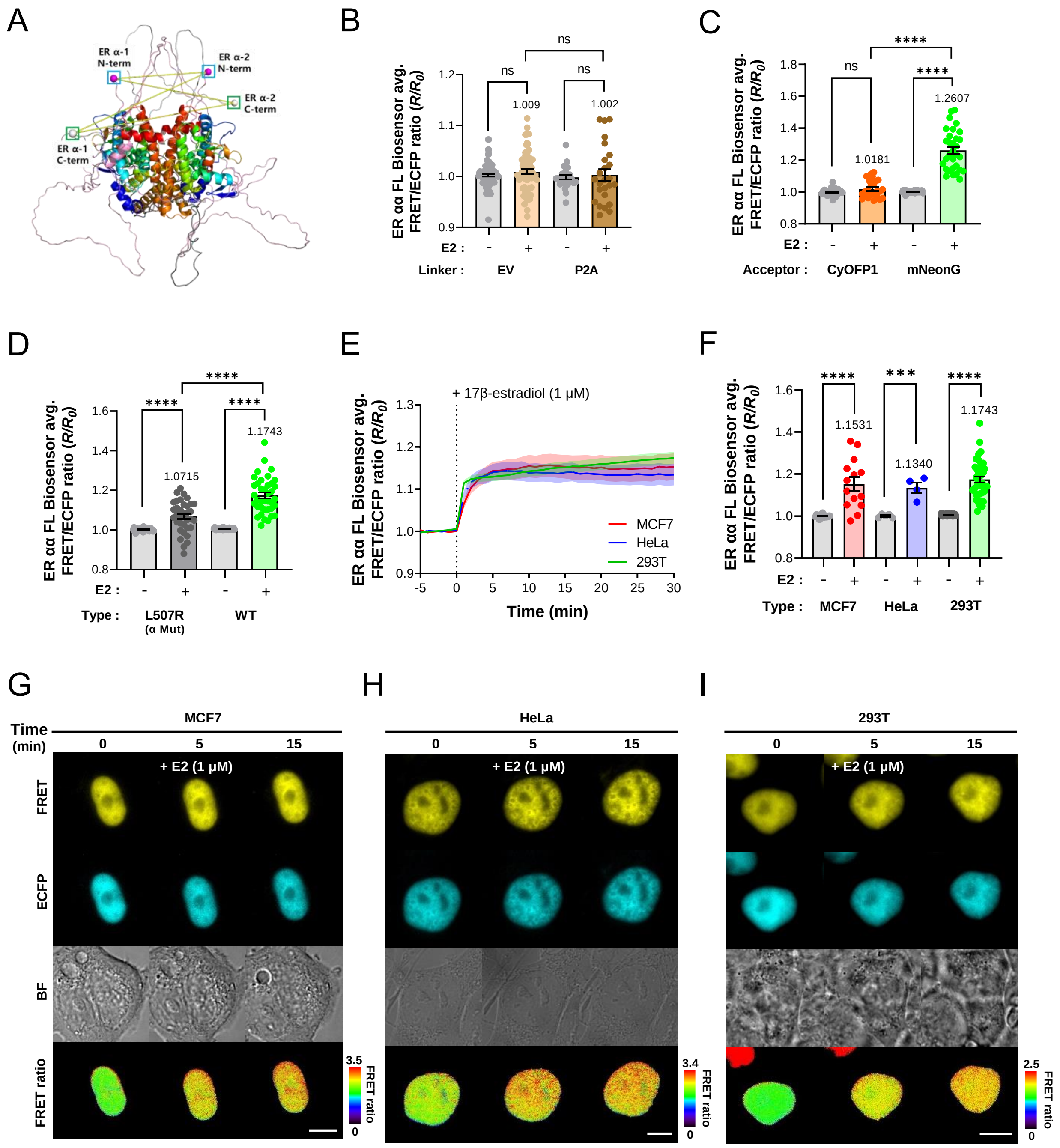

Figure S1. Optimization and Application of the Estrogen Receptor αα Full-Length (FL) FRET Biosensor.

(A) Overall structure of the homodimer of the full-length estrogen receptor (ER) α. The N-terminal regions of each ER are represented by pink circles within blue squares, and C-terminal regions are represented as green squares with white circles. Yellow dashed lines indicate distances between corresponding ends. (B) Bar graphs depict changes in the FRET/ECFP emission ratio between the ER αα FL CyOFP1-EV biosensor (EV; n = 61) and the ER αα FL CyOFP1-P2A biosensor (P2A; n = 25) following treatment with 10 μM 17β-estradiol (E2) for 30 min (ns: not significant). (C) Bar graphs depict changes in the FRET/ECFP emission ratio of the ER αα FL CyOFP1-P2A biosensor (CyOFP1; n = 25) and ER αα FL FRET biosensor

(mNeonGreen; n = 31) following treatment with 10 μM 17β-estradiol for 30 min (****$p < 0.0001$). (D) Bar graphs indicate changes in the FRET/ECFP emission ratio between the ER αα FL FRET biosensor (WT; n = 38) and the ER αα FL L507R (α-mutant) biosensor (L507; n = 35) following treatment with 1 μM 17β-estradiol (E2) for 30 min (****$p < 0.0001$). (E) Time courses and (F) bar graphs of mean changes in normalized FRET/ECFP emission ratio of the ER αα FL FRET biosensor among different cell lines (MCF7; n = 14, HeLa; n = 4, 293T; n = 38, ****$p < 0.0001$, ***$p < 0.001$). (G-I) Time course of FRET, ECFP, bright field, and FRET ratio images of the ER αα FL FRET biosensor using different cell lines, scale bar = 10 μm. All error bars represent mean (line) ± SEM, and all p-values are derived from Student's t-tests.

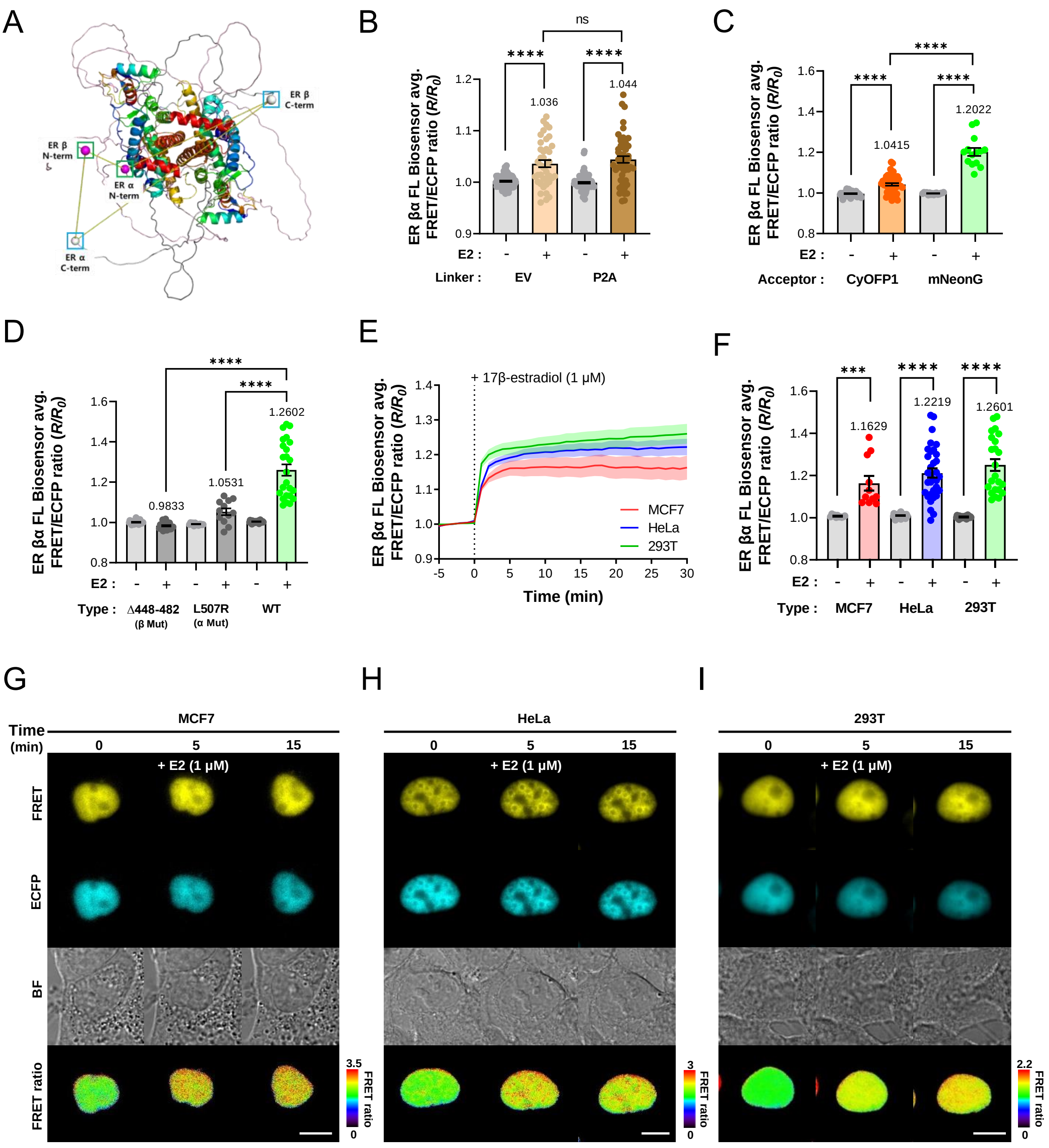

Figure S2. Optimization and Application of the Estrogen Receptor βα Full-Length (FL) FRET Biosensor.
(A) Overall structure of the heterodimer of the full-length ER α and β. (B) Bar graphs show changes in FRET/ECFP emission ratio between the ER βα FL CyOFP1-EV biosensor (EV; n = 38) and the ER βα FL CyOFP1-P2A biosensor (P2A; n = 52) following treatment with 10 μM 17β-estradiol (E2) for 30 min (****p < 0.0001, ns: not significant). (C) Bar graphs depict changes in FRET/ECFP emission ratio of the ER βα FL CyOFP1-P2A biosensor (CyOFP1; n = 49) and the ER βα FL FRET biosensor (mNeonGreen; n = 14) following treatment with 10 μM 17β-estradiol for 30 min (****p < 0.0001). (D) Bar graphs indicate changes in FRET/ECFP emission ratio between the ER βα FL FRET biosensor (WT; n = 23), the ER βα FL L507R (α-

mutant) biosensor (L507; n = 12), and the ER βα FL Δ448-482 (β-mutant) biosensor (Δ448-482; n = 24) following treatment with 1 μM 17β-estradiol (E2) for 30 minutes (****$p < 0.0001$). (E) Time courses and (F) bar graphs of mean normalized changes in FRET/ECFP emission ratio of the ER βα FL FRET biosensor among different cell lines (MCF7; n = 11, HeLa; n = 33, 293T; n = 24, ****$p < 0.0001$, ***$p < 0.001$). (G-I) Time courses of FRET, ECFP, bright field, and FRET ratio images of the ER βα FL FRET biosensor in different cell lines, scale bar = 10 μm. All error bars represent mean (line) ± SEM, and all p-values are derived from Student's t-tests.

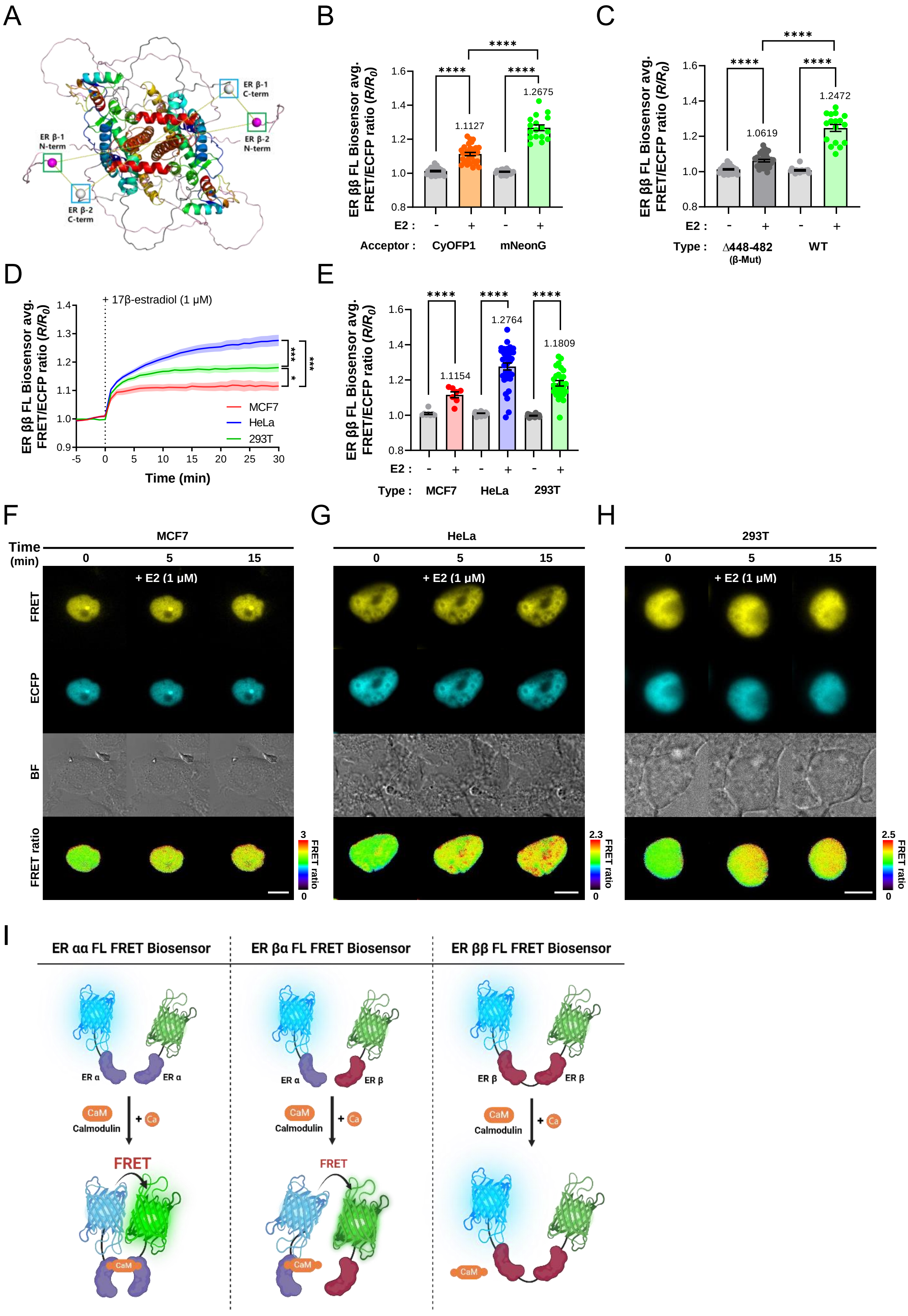
A
ER β-1 C-term
ER β-2 N-term
ER β-1 N-term
ER β-2 C-term
B
ER ββ FL Biosensor avg. FRET/ECFP ratio (R/R0)
1.1127
1.2675
E2 : - + - +
Acceptor : CyOFP1 mNeonG
C
1.0619
1.2472
Type : Δ448-482 (β-Mut) WT
D
+ 17β-estradiol (1 μM)
MCF7
HeLa
293T
Time (min)
E
1.1154
1.2764
1.1809
Type : MCF7 HeLa 293T
F
G
H
Time (min)
+ E2 (1 μM)
FRET
ECFP
BF
FRET ratio
I
ER αα FL FRET Biosensor
ER βα FL FRET Biosensor
ER ββ FL FRET Biosensor
ER α
ER β
CaM
Calmodulin
Ca
FRET

Figure S3. Optimization and Application of the Estrogen Receptor ββ Full-Length (FL) FRET Biosensor.

(A) Overall structure of the homodimer of the full-length ER β. (B) Bar graphs display changes in FRET/ECFP emission ratio for the ER ββ FL CyOFP1-EV biosensor (CyOFP1; n = 31) and the ER ββ FL FRET biosensor (mNeonGreen; n = 17) following treatment with 10 μM 17β-estradiol for 30 min (****$p < 0.0001$). (C) Bar graphs show changes in the FRET/ECFP emission ratio between the ER ββ FL FRET biosensor (WT; n = 17) and the ER ββ FL Δ448-482 (β-mutant) biosensor (Δ448-482; n = 31) following treatment with 1 μM 17β-estradiol (E2) for 30 min (****$p < 0.0001$). (D) Time courses and (E) bar graphs of mean normalized changes in FRET/ECFP emission ratio for the ER ββ FL FRET biosensor among different cell lines (MCF7; n = 7, HeLa; n = 33, 293T, ****$p < 0.0001$, ***$p < 0.001$, *$p < 0.05$). (F-H) Time course of FRET, ECFP, bright field, and FRET ratio images of the ER ββ FL FRET biosensor for different cell lines, scale bar = 10 μm. (I) Schematic representation of the interaction between calmodulin and ER FRET biosensors. All error bars represent mean (line) ± SEM, and all p-values are derived from Student's t-tests.

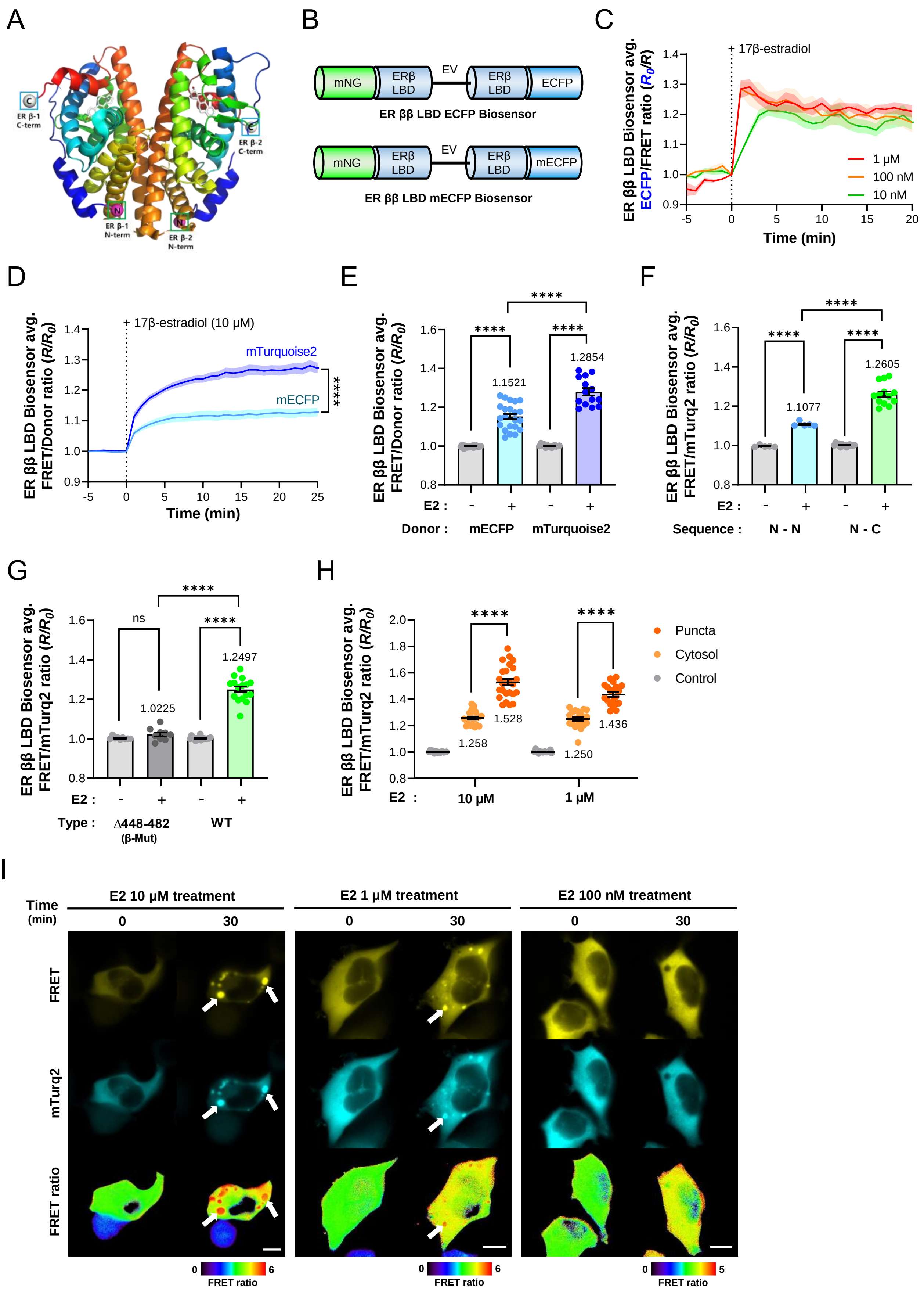
A
ER β-1 C-term
ER β-2 C-term
ER β-1 N-term
ER β-2 N-term
B
mNG
ERβ LBD
EV
ERβ LBD
ECFP
ER ββ LBD ECFP Biosensor
mNG
ERβ LBD
EV
ERβ LBD
mECFP
ER ββ LBD mECFP Biosensor
C
+ 17β-estradiol
ER ββ LBD Biosensor avg. ECFP/FRET ratio (R_0/R)
1 μM
100 nM
10 nM
Time (min)
D
+ 17β-estradiol (10 μM)
mTurquoise2
mECFP
****
ER ββ LBD Biosensor avg. FRET/Donor ratio (R/R_0)
Time (min)
E
ER ββ LBD Biosensor avg. FRET/Donor ratio (R/R_0)
****
1.1521
1.2854
E2 :
Donor :
mECFP
mTurquoise2
F
ER ββ LBD Biosensor avg. FRET/mTurq2 ratio (R/R_0)
****
1.1077
1.2605
E2 :
Sequence :
N - N
N - C
G
ER ββ LBD Biosensor avg. FRET/mTurq2 ratio (R/R_0)
ns
****
1.0225
1.2497
E2 :
Type :
Δ448-482
(β-Mut)
WT
H
ER ββ LBD Biosensor avg. FRET/mTurq2 ratio (R/R_0)
****
1.258
1.528
1.250
1.436
E2 :
10 μM
1 μM
Puncta
Cytosol
Control
I
Time (min)
E2 10 μM treatment
E2 1 μM treatment
E2 100 nM treatment
0
30
FRET
mTurq2
FRET ratio
FRET ratio

Figure S4. Optimization and Feature Analysis of the Estrogen Receptor ββ Ligand-Binding Domain (LBD) FRET Biosensor.

(A) Overall structure of the homodimer of the ligand-binding domain of the Estrogen Receptor ββ. (B) Schematic diagram of the ER ββ LBD ECFP and mECFP biosensors. (C) Time courses of mean normalized changes in ECFP/FRET emission ratio for the ER ββ LBD ECFP biosensor before and after treatment with 17β-estradiol for 20 minutes (1μM; n = 3, 100nM; n = 5, 10nM; n = 6). (D) Time courses and (E) bar graphs show mean changes in normalized FRET/CFP emission ratio for the ER ββ LBD mECFP biosensor (mECFP; n = 22) and the ER ββ LBD FRET biosensor (mTurquoise2; n = 14) before and after treatment with 10 μM 17β-estradiol (****$p < 0.0001$). (F) Bar graphs depict changes in FRET/mTurquoise2 emission ratio of the ER ββ LBD N-N term-EV biosensor (N-N; n = 5) and the ER ββ LBD FRET biosensor (N-C; n = 13) following treatment with 10 μM 17β-estradiol for 30 min (****$p < 0.0001$). (G) Bar graphs show changes in FRET/mTurquoise2 emission ratio of the ER ββ LBD FRET biosensor (WT; n = 16) and the ER ββ LBD Δ194-228 (β-mutant) FRET biosensor (Δ194-228; n = 9) following treatment with 1 μM 17β-estradiol for 30 min (****$p < 0.0001$). (H) Dot graphs show different FRET ratios in cytosolic (10 μM; n = 22, 1 μM; n = 21) and aggregation regions (10 μM; n = 25, 1 μM; n = 20, ****$p < 0.0001$). (I) Time courses of FRET, mTurquoise2, and FRET ratio images of the ER ββ LBD FRET biosensor following treatment with 17β-estradiol for 30 min. White arrows indicate areas of aggregation, scale bar = 10 μm. All error bars represent mean (line) ± SEM, and all p-values are derived from Student's t-tests.

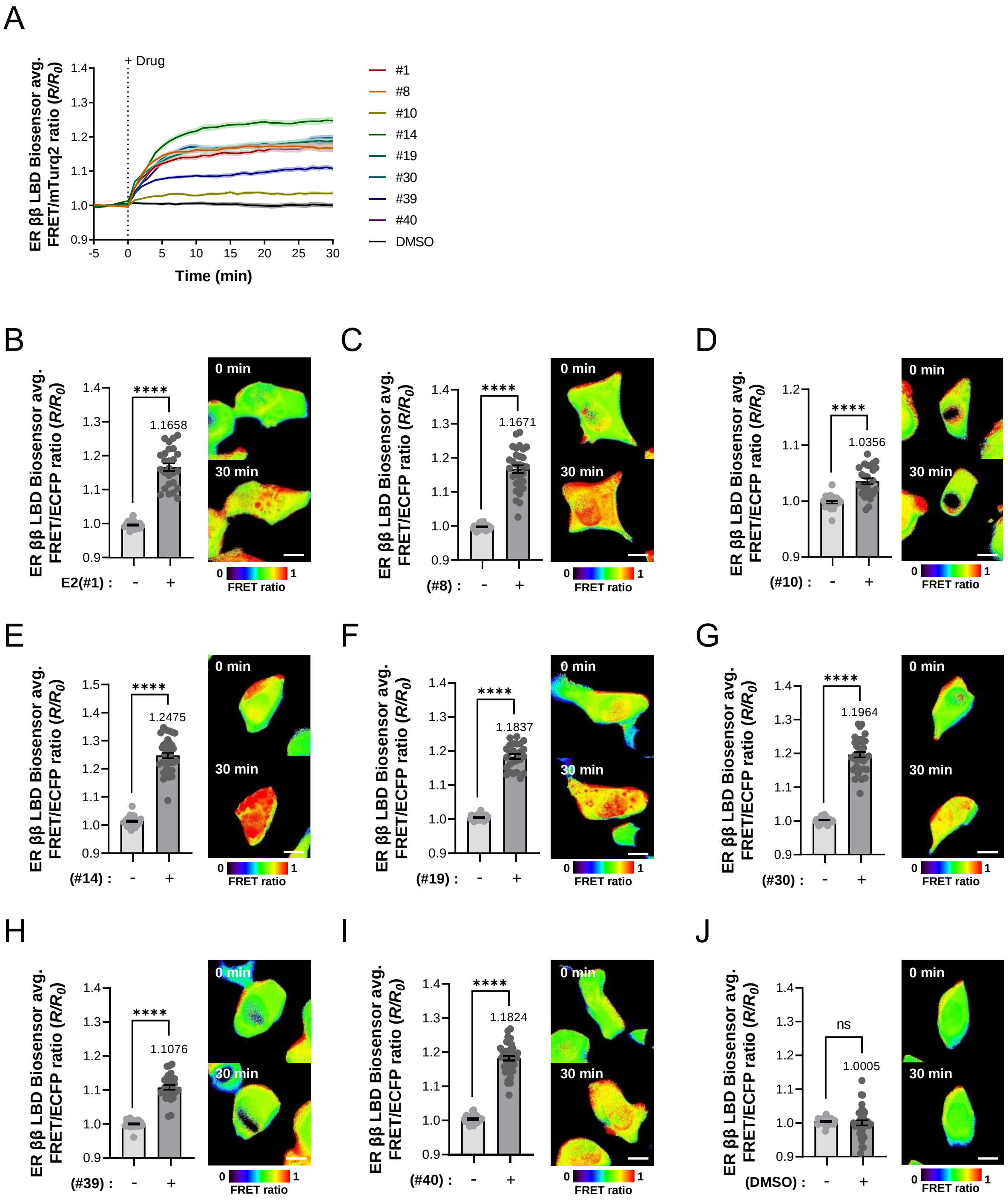

Figure S5. Cross-validation of drug screening results by fluorescence microscopy.
(A) Time course of changes in the mean normalized FRET/mTurquoise2 emission ratio for ER ββ LBD FRET biosensor before and after treatment with 1 μM drugs, which were detected by screening 72 drugs for 30 min. (B-J) Bar graphs (left) and representative images (right) illustrate the FRET ratios of the ER ββ LBD FRET biosensor (#1; n = 26, #8; n = 30, #10; n = 26, #14; n = 35, #19; n = 26, #30; n = 33, #39; n = 29, #40; n = 36, DMSO; n = 36, ****$p < 0.0001$, ns: not significant), scale bar = 10 μm.
